# A functionally selected *Acinetobacter* sp. phosphoethanolamine transferase gene from the goose fecal microbiome confers colistin resistance in *E. coli*

**DOI:** 10.1128/aem.02468-25

**Published:** 2026-08-06

**Authors:** Elizabeth Bernate, Yijun Shi, Ezabelle Franck, Terence S. Crofts

**Affiliations:** 1 College of Veterinary Medicine, University of Florida 3463, Gainesville, Florida, USA; 2 Department of Animal Sciences, University of Illinois at Urbana-Champaign 14589, Urbana, Illinois, USA; 3 Department of Biomedical Sciences, Florida State University 7823, Tallahassee, Florida, USA; 4 Carl R. Woese Institute for Genomic Biology, University of Illinois at Urbana-Champaign 14589, Urbana, Illinois, USA; 5 Division of Nutritional Sciences, University of Illinois at Urbana-Champaign 14589, Urbana, Illinois, USA; Universidad de los Andes, Bogotá, Colombia

**Keywords:** MCR, EptA, functional metagenomics, colistin, antibiotic resistance

## Abstract

Polymyxins are last-resort antibiotics for infections caused by multidrug-resistant gram-negative bacteria such as Enterobacteriaceae, *Pseudomonas aeruginosa,* and *Acinetobacter baumannii*. This makes the rise of bacteria exhibiting polymyxin E (colistin) resistance, largely through modification of lipid A moieties, concerning and suggests that it is important to document the potential sources of the corresponding resistance genes. This study searched for potential emerging colistin resistance genes from the environment by investigating a previously performed functional metagenomic selection for colistin resistance of a goose fecal microbiome. We found that the selection captured *Acinetobacter* sp. DNA fragments that all contained *eptA* genes. We confirmed their ability to confer significant colistin resistance in *Escherichia coli* via modification of lipid A in the outer membrane. Furthermore, we found evidence for mobilization of closely related *eptA* genes in *Acinetobacter* genomes, marking them as potential *mcr* genes or their precursors. This study highlights the potential for functional metagenomic selections for colistin resistance to capture genes from unexpected environmental sources such as the goose fecal microbiome.

**IMPORTANCE:**

Colistin is an important antibiotic of last resort, and increasing resistance to this drug via mobile phosphoethanolamine transferase genes, such as *mcr-1*, threatens its clinical utility. Given the discovery of *mcr-1* in pigs, the ability of animals to act as vectors in the spread of colistin resistance is alarming. We show here that functionally selected *Acinetobacter* phosphoethanolamine transferase genes from the goose microbiome have the ability to confer clinical levels of colistin resistance when transferred into *E. coli*. While the genes are annotated as *eptA* homologs, closer study of these genes suggests that they may be mobilized within the *Acinetobacter* genus, suggesting that they may be *mcr* genes of concern instead.

## INTRODUCTION

Polymyxin antibiotics were discovered and isolated in 1947 by Y. Koyama from the soil bacterium *Paenibacillus polymyxa* subspecies *colistinus* (1). As antibiotics, polymyxins were initially disregarded due to their harmful side effects compared to other antibiotic classes, but they were eventually adopted into the clinic due to their ability to treat otherwise antibiotic-resistant gram-negative pathogens. The only commercially available compounds from this class are polymyxin B and polymyxin E (also known as colistin) (2). Polymyxin B and colistin are both peptide antibiotics and differ from each other by only one amino acid and have similar microbiological activity (3). Polymyxin B is used topically, while colistin can be used to treat systemic infections as well (4). Colistin has two different commercially available forms: colistin sulfate as an oral and topical medication and colistimethate, which can be administered via injection (5). Colistin was first approved and used in the 1950s against otherwise resistant gram-negative bacterial infections and was praised for its comparatively low level of antibiotic resistance in both human and veterinary settings (1). In the 1970s, colistin became less frequently used in human medicine due to its side effects of nephrotoxicity and neurotoxicity (6) but was reintroduced into the clinic in the 1990s (7) to treat cystic fibrosis-associated gram-negative infections. Colistin now serves as a last line of defense against multidrug-resistant infections. In contrast to human medicine, colistin has been continuously and extensively used in veterinary medicine (8) for the treatment of diseases caused by bacteria such as *E. coli* and *Salmonella* in rabbits, poultry, and livestock (9) and *Aeromonas* and *Shewanella* in aquaculture (10, 11). In agricultural settings (mainly swine), colistin is or has been given orally through drinking water or feed for multiuse infection prevention and growth promotion (12). Colistin is active against many gram-negative bacteria, and there are concerns that its prophylactic use in agriculture, leading to the release of colistin into other environments, contributes to increased selection for bacterial resistance (8, 13).

Colistin is an acylated cyclic polypeptide that is composed of over 30 polymyxin congeners, mainly polymyxin E1 and polymyxin E2, and operates by interacting as a net positively charged structure that binds to the negative phosphates of the lipid A lipopolysaccharide (LPS) of gram-negative bacteria. Binding of colistin to LPS destabilizes the calcium and magnesium bridges on the outer membrane via hydrophobic and electrostatic interaction (14). This destabilization causes the release of the LPS and permeabilizes the outer membrane, allowing the antibiotic to pass to the cytosolic membrane through the lipophilic acid-fat chain where it causes disruption of the phospholipid bilayer, resulting in osmotic disparity between the inside and outside of the cell and, eventually, lysis (1, 15). Other mechanisms may be involved in its antibacterial activity, such as inhibition of respiratory enzymes (16) and induction of oxidative stress caused by an imbalance of oxygen reactive species (17).

The dominant mechanism of colistin resistance acts by preventing binding of the drug to its lipid A target. This can occur through the transfer of cationic moieties onto lipid A, either by a phosphoethanolamine transferase (e.g*.,* MCR and EptA) or an aminoarabinose transferase (e.g*.,* ArnT). This outer membrane modification results in an increased density of positive charges on the cell membrane and decreases the binding affinity of colistin (Fig. S1). Additionally, chromosomal mutations affecting synthesis of lipid A or increased lipid A 4′-phosphatase activity can also cause a decrease in negatively charged phosphates in the outer membrane, ultimately decreasing the electrostatic interaction and affinity of colistin to bind and affect the bacteria (7, 18–20). Additional mechanisms of colistin resistance exist that do not rely on lipid A modification. These include active efflux of colistin and biofilm formation, both of which decrease the amount of colistin that reaches the cytosolic membrane (2, 21, 22).

Colistin resistance can also arise from a regulatory two-component system PmrAB (coupled to PhoP/Q by PmrD) that upregulates lipid A modification operons (6, 23, 24). Acquired resistance can be due to mutations or changes in the genomic context of this system that trigger transcription of lipid A modification genes. More concerning are mobilized colistin resistance mechanisms. In 2015, as part of an antimicrobial resistance monitoring project in China, a colistin-resistant *E. coli* isolate (strain SHP45) was isolated from a pig (3). Resistance was determined to be conferred by a gene carried on a plasmid. Because the gene could potentially be mobilized to other bacteria via the plasmid, it was termed *mcr*-1 for mobilizable colistin resistance. The *mcr* gene family encodes phosphoethanolamine transferases, similar to those encoded by the *eptA* gene family, which confer resistance through charge alteration in lipid A (25–27) (Fig. S1). Studies have since found evidence for *mcr* genes in *E. coli* isolates from as far back as the 1970s (28) and in a variety of plasmid contexts and bacterial hosts (29), suggesting their spread across environments.

The early emergence of *mcr* and its characteristic of transmissibility suggest that there are homologs in the natural environment yet to be found. This is born-out by the rapid discovery of *mcr* genes 2 through 10 (30). Notably, while approximately half of biomedical literature focuses on just ten readily cultured, largely pathogenic taxa, resistance genes from non-model or non-clinical organisms are of great importance, especially as potential reservoirs of antimicrobial resistance (31, 32). Here, we used a functional metagenomics approach to search for potential colistin-resistance genes from the fecal microbiome of a wild goose. Functional metagenomics is a One-Health compatible technique that allows for sequence-naïve, high-throughput identification of genes from an entire microbiome’s metagenome based on their ability to confer antibiotic resistance. Functional metagenomic libraries are prepared by extraction of metagenomic DNA from a microbiome (in our case, a goose fecal pellet), fragmentation of metagenomic DNA, cloning of the random metagenomic DNA fragments into a plasmid, and transformation of an *E. coli* expression host with the plasmid library (Fig. S2). This *E. coli* library is then spread onto agar plates containing inhibitory concentrations of antibiotics (e.g*.,* 4 µg/mL colistin), and plasmid-contained metagenomic fragments from resistant colonies are sent for sequencing (Fig. S2). This technique can identify both known and novel antibiotic resistance genes as well as potentially identify evidence of horizontal transfer of antibiotic resistance genes (33, 34).

## MATERIALS AND METHODS

### Bacterial strains, reagents, and chemicals

Cultivation of *E. coli* (DH10B genotype) was performed at 35°C, with aeration by shaking at 250 rpm for liquid cultures, unless otherwise specified. This incubation temperature was chosen to conform to EUCAST antimicrobial resistance testing recommendations, which avoid growth at 37°C in order to prevent accidental overshoot into inhibitory temperatures (35). Routine cultures were grown in lysogeny broth (Miller) (LB) with 50 μg/mL kanamycin (LB + KAN 50) for maintenance of plasmids. Antimicrobial susceptibility testing was carried out using Cation-Adjusted Mueller-Hinton media (MH) (Teknova, 101320-364) with 50 μg/mL kanamycin (MH + KAN 50) when appropriate. Plasmids used in this study were derivatives of pZE21 unless otherwise noted (33, 36, 37). Bacterial clones were stored in a −80°C freezer as 15% glycerol stocks in LB.

The *mcr*-expressing positive control *E. coli* strain used during antimicrobial susceptibility testing and lipid A mass spectrometry analysis was from the Minimal Antibiotic Resistance Platform (ARP) and a gift from Gerard Wright (Addgene kit #1000000143) (38).

Reagents and chemicals were of molecular biology grade or higher purity. Kanamycin sulfate (VWR Life Science, 75856-68) and colistin sulfate (Sigma Aldrich, C44611G) powders were stored at 4°C in a desiccator while solutions were stored at −20°C as sterile filtered 50 mg/mL stock solutions. Minimal inhibitory concentration test strips for polymyxin B (cat# 920041) and colistin (cat# 921411) were purchased from Liofilchem.

### Functional metagenomic selection

A goose fecal microbiome functional metagenomic library was previously constructed and selected for colistin resistance (33). Briefly, a freshly voided fecal pellet from a mature Canada goose (*Branta canadensis*) was collected from a grass lawn on the Northwestern University Evanston campus lakefill (coordinates: 42°03′21.9″N, 87°40′14.4″W) in August 2020, and metagenomic DNA extraction was initiated within 10 min of collection. The metagenomic DNA extraction protocol was based on the Qiagen DNeasy PowerSoil kit (Qiagen, catalog no. 12888-100) with extended incubation in buffer CD1 at 65°C (10 min) and at 95°C (10 min) (33, 39). The extracted metagenomic DNA was used to prepare a functional metagenomic library by the METa assembly protocol (33). The resulting functional metagenomic library was estimated to contain ~1.2 × 10^8^ unique clones with 95.2% of clones predicted to have captured a metagenomic DNA fragment with an average size of 2.4 kb. In total, the library was estimated to encode ~27 Gb of metagenomic DNA (33).

The library was plated onto Mueller-Hinton cation-adjusted II (MH) agar plates containing either 4 μg/mL or 8 μg/mL colistin sulfate and incubated overnight in a shared instrument set at 37°C. Initially, no resistant colonies were observed on the 8 μg/mL agar plate, but two were found after an additional incubation at room temperature, which were saved for analysis. The agar plates containing 4 μg/mL colistin resulted in an estimated 360 resistant colonies after incubation overnight. The resistant colonies were collected from the agar plate by resuspending them with a cell spreader in 1 mL of LB (4 μg/mL selection) or directly picking into LB + KAN 50 media and culturing (8 μg/mL colonies). Plasmids were isolated from the resulting slurry or cultures via miniprep (New England Biolabs, T1010L) according to the manufacturer’s protocols and stored at −20°C until sequencing.

### Metagenomic fragment sequencing and processing

Colistin resistance-conferring metagenomic fragments from the pooled slurry of the 4 μg/mL colistin plate colonies were previously sequenced (33). Briefly, the metagenomic inserts were amplified by PCR from the slurry miniprep using Q5 polymerase (New England Biolabs, M0492S) in a 100-μL reaction with 5 ng of DNA as the template using primers that amplify from 250 bp either side of the vector mosaic end sequence sites. The thermocycler settings included an initial 30-s hold at 98°C, followed by ten cycles of 98°C for 10 s and 72°C for 4 min. The amplified metagenomic inserts were purified by a silica column kit (New England Biolabs, T1030S) and quantified by fluorescence (Promega QuantiFluor, E4871). The resulting purified colistin resistance-conferring amplicons were submitted for sequencing on the PacBio Sequel II platform at the Roy J. Carver Biotechnology Center at the University of Illinois at Urbana-Champaign.

For analysis, the PacBio reads were first processed in Galaxy to remove vector sequences from the reads (40). The trimmed reads were dereplicated using cd-hit-est with a 99% nucleotide identity cut-off and otherwise default settings (41–43). The resulting clusters were sorted by the number of reads in the cluster, and the representative read from each was extracted for analysis. Metagenomic fragments from the 16 highest rank clusters were selected for cloning.

### Cloning of metagenomic fragments

The targeted metagenomic DNA fragments from the 4 µg/mL functional metagenomic selection were amplified and cloned into a pZE21 derivative (36) with a modified cloning site 8 bp downstream of the vector ribosome-binding site (pTSC174) (37). Amplification was carried out using Q5 polymerase with a 2-step PCR protocol (New England Biolabs, M0492S), and inserts were cloned into pTSC174 using NEBuilder HiFi assembly mix (New England Biolabs, E2621L) according to the manufacturer’s protocols. Chemically competent *E. coli* DH10B cells were transformed by the recombinant plasmids using heat shock (44), and plasmids from the transformed *E. coli* were analyzed by full plasmid sequencing through Plasmidsaurus. Plasmids extracted from the two *E. coli* clones that grew on the 8 μg/mL colistin plates were sequenced without re-cloning.

### Bioinformatic analysis

Analysis of the gene contents of the metagenomic inserts was performed by calling open reading frames and annotating the resulting predicted proteins using the Bakta server (45, 46), MetaGeneMark (47), and by using blastp to align the predicted proteins against non-redundant protein sequences (conducted October, 2025) (48, 49). Relative coordinates for the predicted genes were used to construct gene schematics.

The EptA predicted to be encoded on the 8 μg/mL selected metagenomic DNA fragment (EptA17) was compared to representative EptA, EptB, EptC, and MCR protein sequences. UniProt 50% Reference Clusters (50, 51) were downloaded for EptA, EptB, and EptC. MCR sequences (ARO:3004268) were downloaded from the Comprehensive Antibiotic Resistance Database (CARD) (52, 53). Protein sequences were aligned using the Clustal Omega webserver (54), and the alignment was used to construct a maximum likelihood tree with IQ-Tree (VT + R 6 model, 100 replicates) (55, 56). Redundant enzymes and those lacking genus-level annotation were removed, and the tree was visualized with the Interactive Tree of Life (iTOL) program (57).

For the comparison of EptA17 against *Acinetobacter* taxa, the predicted amino acid sequence was used as the query in a regular protein BLAST alignment with default parameters against the following taxa: *A. albensis* (taxid:1673609), *A. apis* (taxid:1229165), *A. baumannii* (taxid:470), *A. baylyi* (taxid:202950), *A. beijerinckii* (taxid:262668), *A. bereziniae* (taxid:106648), *A. boissieri* (taxid:1219383), *A. bouvetii* (taxid:202951), *A. brisouii* (taxid:396323), *A. calcoaceticus* (taxid:471), *A. celticus* (taxid:1891224), *A. colistiniresistens* (taxid:280145), *A. courvalinii* (taxid:280147), *A. dispersus* (taxid:70348), *A. defluvii* (taxid:1871111), *A. dispersus* (taxid:70348), *A. equi* (taxid:1324350), *A. gandensis* (taxid:1443941), *A. gerneri* (taxid:202952), *A. guillouiae* (taxid:106649), *A. gyllenbergii* (taxid:134534), *A. haemolyticus* (taxid:29430), *A. harbinensis* (taxid:1353941), *A. indicus* (taxid:756892), *A. johnsonii* (taxid:40214), *A. junii* (taxid:40215), *A. kookii* (taxid:1226327), *A. lactucae* (taxid:1785128), *A. larvae* (taxid:1789224), *A. lwoffii* (taxid:28090), *A. modestus* (taxid:1776740), *A. nectaris* (taxid:1219382), *A. nosocomialis* (taxid:106654), *A. oleivorans* (taxid:1148157), *A. parvus* (taxid:134533), *A. piscicola* (taxid:2006115), *A. pittii* (taxid:48296), *A. populi* (taxid:1582270), *A. proteolyticus* (taxid:1776741), *A. radioresistens* (taxid:40216), *A. rudis* (taxid:632955), *A. schindleri* (taxid:108981), *A. seifertii* (taxid:1530123), *A. sichuanensis* (taxid:2136183), *A. soli* (taxid:487316), *A. tandoii* (taxid:202954), *A. tjernbergiae* (taxid:202955), *A. towneri* (taxid:202956), *A. ursingii* (taxid:108980), *A. venetianus* (taxid:52133), *A. vivianii* (taxid:1776742), *A. wuhouensis* (taxid:1879050), and *A. stercoris* (taxid:2126983). The resulting alignment was used to build a neighbor-joining tree using the built-in BLAST tree viewer and visualized using the iTOL program (57).

Comparison of the composite *cst* nucleotide sequence to other *Acinetobacter* genomes was conducted in two stages. In the first stage, we aligned the *cst* sequence against the NCBI core nucleotide collection (core nt) using megablast. In the second stage, we aligned the *cst* sequence against genomes of specific *Acinetobacter* species (see list above) using the discontinuous megablast algorithm. The resulting hits were ranked by overall coverage to find the genome with the highest coverage to the *cst* nucleotide sequence (58). Genomic DNA sequences from approximately 12 kb upstream and downstream of the aligned DNA sequence were extracted and searched for antibiotic resistance genes using the CARD Resistance Gene Identifier (RGI) tool (52, 53) and were manually scanned for genes with annotations associated with genetic mobilization.

### Antimicrobial susceptibility tests

Agar dilution antimicrobial susceptibility assays were carried out as published in Wiegan et al. (59). Briefly, in triplicate, single colonies from each strain were resuspended in 200 μL of MH + KAN 50 broth to reach an OD_600_ at 1 cm of between 0.08 and 1 absorbance units (equivalent to a 0.5 McFarland standard) to produce a bacterial suspension of ~1 × 10^8^ colony-forming units (CFUs) per mL. Suspensions were diluted 10-fold with MH broth in a 96-well plate. A 48-pin microplate replicator (Fisher Scientific, 05 - 450-10), sterilized by dipping into 70% ethanol and flame-dried, was placed in the wells to pick up 1 µL of each culture and stamped onto agar plates composed of MH + KAN 50 and the concentration of colistin being tested (0 µg/mL and 0.03125 μg/mL to 32 μg/mL by 2-fold increments). The agar plates were incubated at 35°C overnight and photographed and analyzed following 20 to 24 h of growth.

Liquid culture 50% inhibitory concentration (IC_50_) values for colistin were determined using broth microdilution assays (35, 59). A 96-well plate was prepared with each well containing 50 μL of MH + KAN 50 and colistin. Colistin concentrations varied by two-fold increments across 10 columns, with the highest colistin concentration being 128 μg/mL. Two control volumes did not contain colistin (column 11 as a growth control and column 12 as a sterility control). A 0.5 McFarland standard for each of the tested strains was prepared as before and diluted 100-fold before 50 μL aliquots were added to the antibiotic-containing 96-well plate to inoculate the wells. The plate was sealed with a Breathe-Easy membrane (MilliporeSigma, Z380059) and incubated at 35°C with shaking at 250 rpm overnight for 20 h to 24 h. Growth was quantified by measuring OD_600_ absorbance on a plate reader (Agilent Biotek, Synergy HTX), and dose-response curves were generated to compute IC_50_ values using GraphPad Prism (version 10.6.1).

Liofilchem colistin and polymyxin test strips were used to find MIC values according to the manufacturer’s instructions. Briefly, colonies of the strains being analyzed were resuspended in MH broth to produce a 0.5 McFarland standard as above. A sterile cotton-tipped swab was dipped into the suspension and used to inoculate the full surface of MH + KAN 50 agar plates. Antibiotic test strips, one for colistin and one for polymyxin B, were placed on the plates. The cultures were incubated at 35°C for 20 h to 24 h, after which they were photographed and analyzed to determine MIC values.

### Mass spectrometry analysis of lipids

Lipid A molecules were extracted from *E. coli* cultures grown overnight at 35°C with shaking at 250 rpm in LB + KAN 50 following the sodium acetate method described by Liang et al. (60). Cultures (1 mL) were pelleted by centrifugation at 8,000 rcf for 1 min, and supernatants were removed. The pellets were resuspended in 400 μL of 100 mM, pH 4 sodium acetate buffer and held at 99°C for 30 min with mixing by briefly vortexing every 10 min. The samples were brought to room temperature by placing on ice and then centrifuged at 8,000 rcf for 5 min. The insoluble pellet was washed once with 95% ethanol, following which lipids were extracted from the washed pellet by addition of 100 μL of a chloroform/methanol/water mix (12:6:1 by volume). The insoluble material in the lipid extract was removed by centrifugation at 5,000 rcf for 5 min. The clarified lipid solution was characterized by matrix-assisted laser desorption/ionization time-of-flight mass spectrometry using a Bruker UltrafleXtreme MALDI TOF/TOF instrument in negative reflector mode at the University of Illinois at Urbana-Champaign School of Chemical Sciences Mass Spectrometry Laboratory.

## RESULTS

### Characterization of colistin resistance-conferring DNA fragments from a functional metagenomic selection of the goose fecal microbiome

We set out to characterize colistin resistance genes from the goose fecal microbiome using functional metagenomic selections. We had previously selected a 27-Gb functional metagenomic library prepared from goose fecal metagenomic DNA on agar plates containing 4 μg/mL colistin sulfate and collected approximately 360 resistant colonies from the selection (33). The sampled goose fecal pellet was collected from a lake-adjacent grassy strip on the Northwestern University campus near where students frequently gather. If the goose fecal microbiome contains ~2,500 taxa (61) with an average genome size of 4 Mb, the total ~10 Gb microbiome is likely to be well represented in this library.

We sequenced the resistance-conferring metagenomic inserts and clustered them at 99% nucleotide identity, resulting in 5576 clusters, the majority of which contained a single read (Fig. S3). After stack ranking the clusters by the number of reads, we focused on the top 16 largest clusters for analysis, which accounted for 68% of all clustered reads (CST1-CST16). We added an additional metagenomic DNA fragment to the analysis that originated from an extended selection of the same library on an agar plate containing 8 μg/mL colistin sulfate (CST17).

On analysis, the metagenomic insert sequencing reads appeared to have quality issues, with most annotation attempts resulting in the calling of fragmented open reading frames, which we hypothesized to most likely be whole.

**Fig 1:**
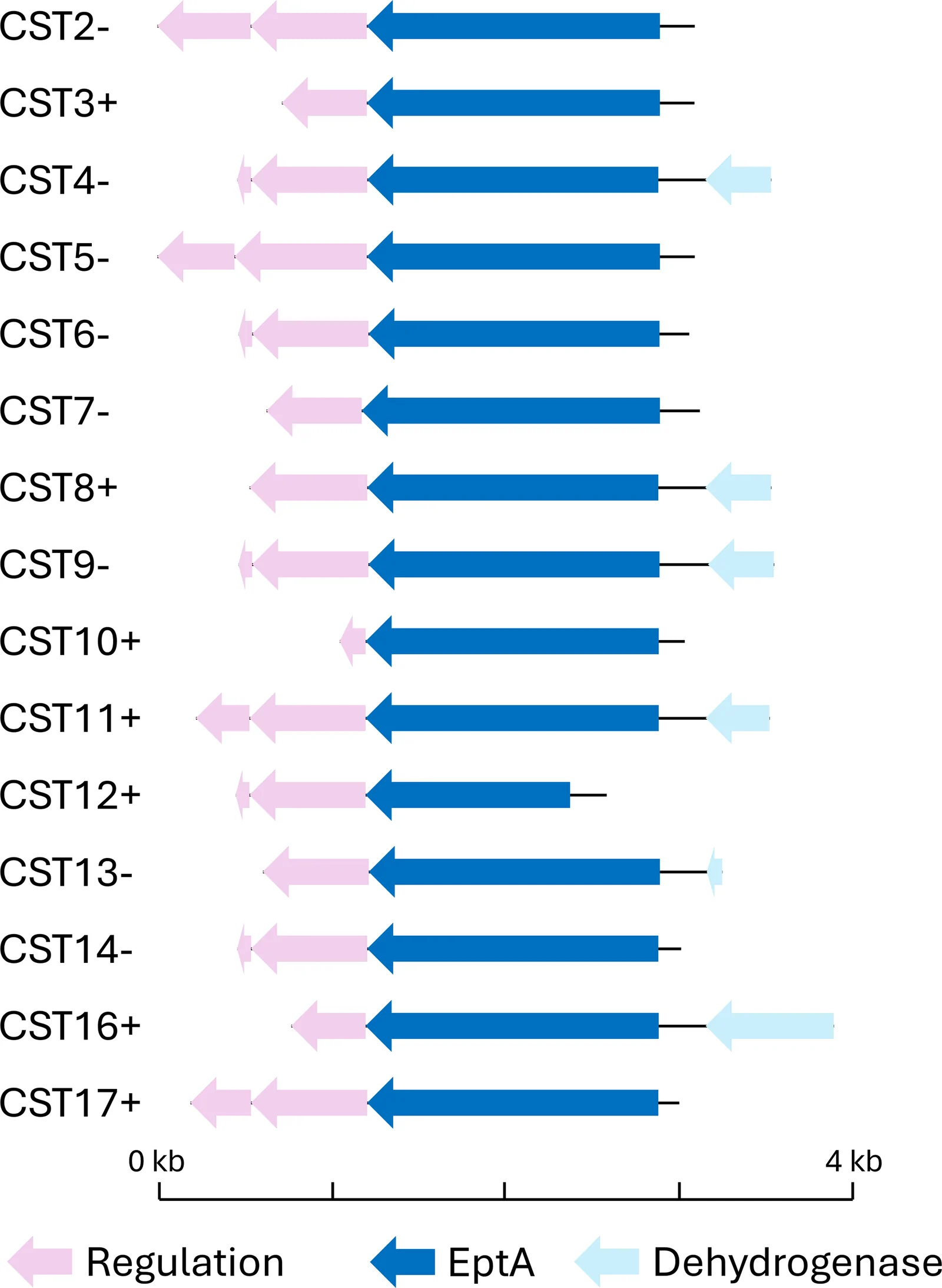
Gene schematics of colistin resistance-conferring metagenomic DNA fragments. Predicted open reading frames from DNA selected by 4 μg/mL (CST2-16) or 8 μg/mL (CST17) colistin selections. Annotations of predicted genes or their fragments include those likely to be related to transcriptional regulation (pink), eptA phosphoethanolamine transferase genes (blue), and a predicted alcohol dehydrogenase gene (light blue). The orientation of the cloned eptA gene with respect to the vector promoter is denoted by “+” or “−” next to the fragment name.

We set out to re-clone these 16 metagenomic inserts. Attempts to clone CST1 and CST15 failed, resulting in 15 total clones, including the 8 μg/mL clone (CST17). We re-sequenced the cloned inserts by long-read technology and analyzed them by blastn of the core nucleotide database and found all of them to have high nucleotide identity to *Acinetobacter* sp. ASP199 (Table S1). The inserts showed a high level of identity to each other (97.62% average nucleotide identity across inserts, Fig. S4A) and a high level of clustering (Fig. S4B) (with CST12 appearing to have acquired a 3′ truncation), suggesting to us that all 15 inserts represent independent fragmentation and library capture events of the same original genomic DNA. After predicting genes on the metagenomic inserts and annotating the resulting open reading frames, it became clear that they all contained a similar genomic construction: one to two genes from an apparent two-component regulatory system (a predicted sensor histidine kinase and response regulator transcription factor), a phosphoethanolamine transferase gene, and, in some cases, a gene predicted to encode an alcohol dehydrogenase gene (Fig. 1), suggesting *eptA* as the resistance mechanism.

**Fig 2:**
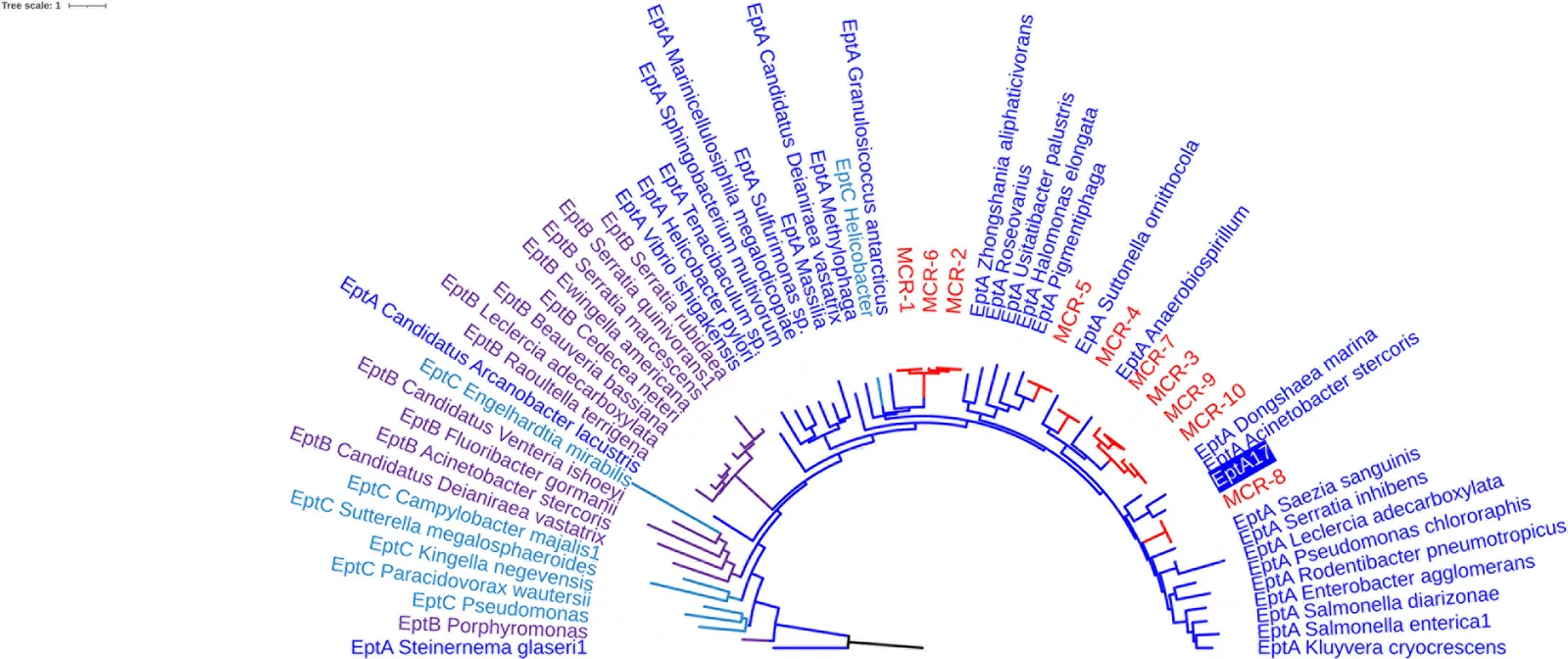
EptA, EptB, EptC, and MCR protein phylogenetic tree. Representative phosphoethanolamine transferase protein sequences from UniRef (EptA, EptB, and EptC) or CARD (MCR) and the predicted EptA from metagenomic DNA fragment CST17 (EptA17). EptA sequences are shown in blue, EptB in purple, EptC in cyan, and MCR in red. EptA17 is highlighted with a blue background.

Using the predicted sequence of the EptA enzyme from the metagenomic fragment selected by growth on 8 μg/mL colistin (EptA17 from CST17), we constructed a phylogenetic tree containing homologs of EptA, MCR, EptB, and EptC (Fig. 2). EptA17 clusters with the EptA protein from *Acinetobacter stercoris* and, more generally, with the branches of the tree associated with EptA and MCR proteins. This situates the enzyme within the phosphoethanolamine transferase family, though it is not clear if it is an EptA or MCR member of this family.

To improve the functional and phylogenetic context of EptA17, we next focused just on EptA homologs from *Acinetobacter* taxa. We performed a protein BLAST alignment of EptA17 against 52 *Acinetobacter* taxa and used the results to prepare a phylogenetic tree (Fig. S5). In this tree, EptA17 clusters with homologs from a mix of *Acinetobacter* taxa including *A. baumannii*, *A. schindleri*, and *A. towneri*.

### *Acinetobacter* sp. *eptA* expression in *E. coli* confers clinically relevant levels of colistin resistance

**Fig 3:**
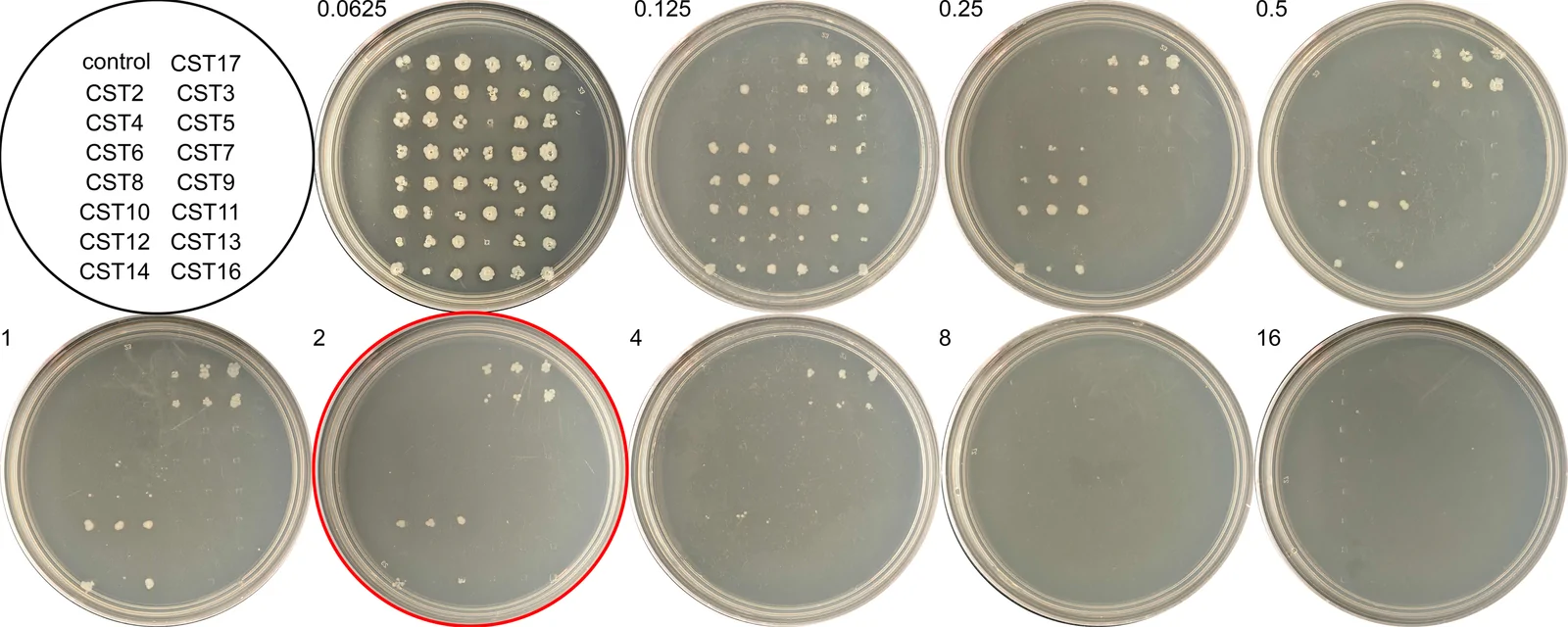
*eptA*-containing metagenomic DNA from *Acinetobacter* sp. decreases colistin susceptibility in *E. coli*. Agar dilution assay plates with 15 eptA-containing clones (CSTs) and one plasmid control (control) spotted in triplicate on agar plates containing colistin at the indicated concentrations (μg/mL). The 2 μg/mL plate is highlighted with a red circle.

We next performed agar dilution colistin susceptibility testing of the 15 functional metagenomic clones and a negative control *E. coli* strain. Except for CST4 and CST9, all of the clones showed a decrease in colistin susceptibility compared to the plasmid-only control (Fig. 3). Four clones, CST3, CST10, CST14, and CST17, grew at colistin concentrations greater than or equal to 2 μg/mL, with clone CST17 in particular showing robust growth at up to 4 μg/mL colistin. Of the clones showing the highest colistin resistance, three (CST3, CST10, and CST17) maintain their *eptA* gene in the same direction as the plasmid promoter and one (CST14) is oriented in the opposite direction (Fig. 1).

**Fig 4:**
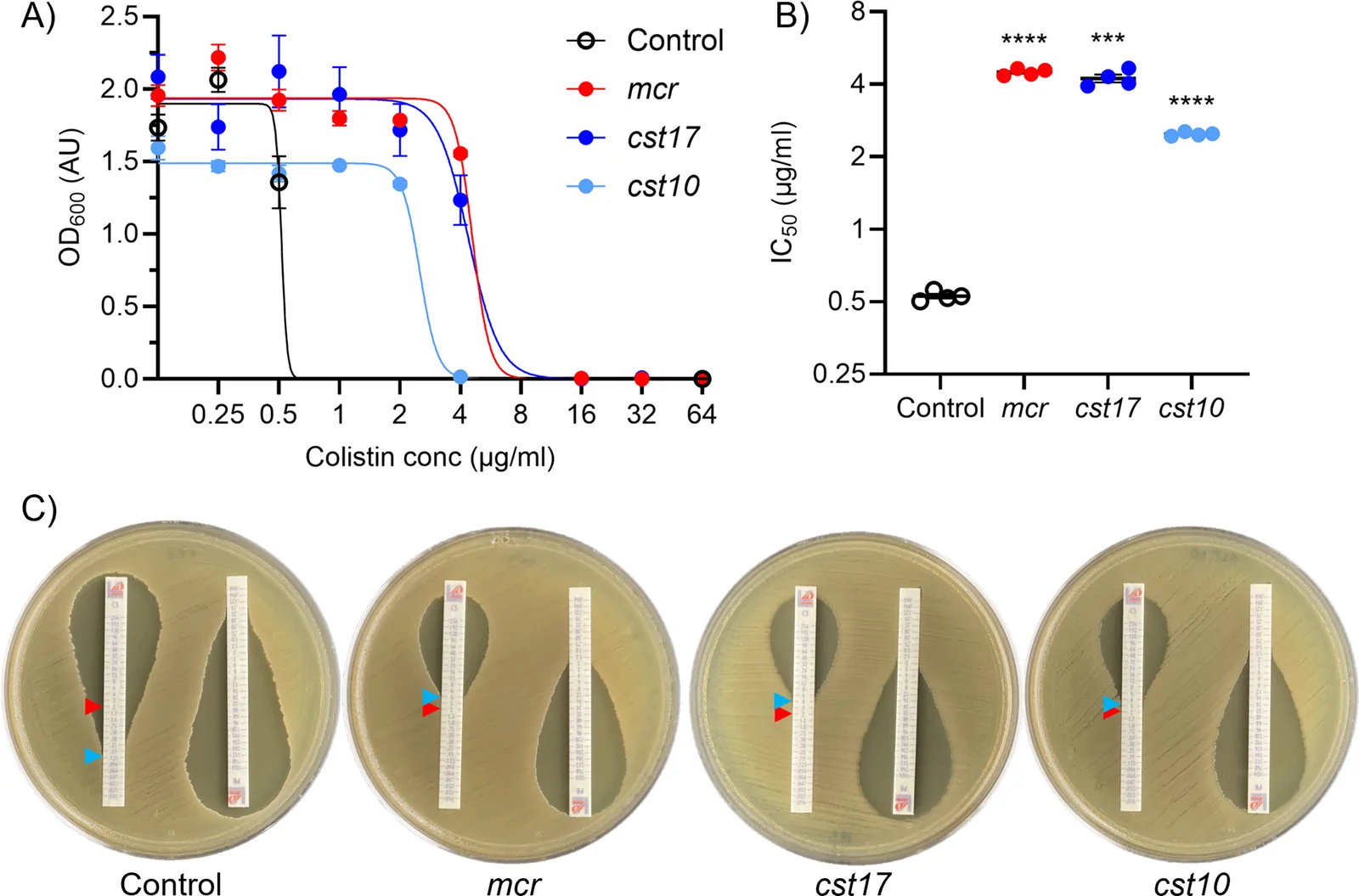
CST10 and CST17 confer colistin resistance in *E. coli*. (**A**) Dose-response curve showing growth of *E. coli* harboring an empty plasmid (control, black) or plasmids containing functionally selected colistin-resistant metagenomic DNA fragments (CST10 and CST17, light and dark blue, respectively) or a bona fide mcr-1 gene (mcr, red). (**B**) 50% inhibitory concentration (IC50) values calculated from the dose-response curves for the same strains (*** *P* = 0.0005 and **** *P* < 0.0001 compared to control). For panels A and B, *n* = 4 replicates with standard error bars. (**C**) Minimal inhibitory concentration (MIC) test strip assays for colistin (left strip) or polymyxin B (right strip) for the indicated strains. Red arrows indicate the 2 μg/mL breakpoint for colistin resistance, and blue arrows indicate the observed MIC.

We next decided to investigate colistin resistance of two of the *E. coli* clones from the selection: CST17 (with *eptA*17 encoded on its captured metagenomic DNA fragment), because it originated from a more stringent 8 μg/mL colistin selection, and CST10, because its relatively short metagenomic DNA fragment is almost completely composed of the predicted *eptA* gene (Fig. 1). The two clones, alongside an empty plasmid negative control and a positive control expressing *mcr*-1, were assayed by microbroth dilution (Fig. 4A). The dose-response curves were solved to determine 50% inhibitory concentration (IC_50_) values (Fig. 4B). The functionally selected clones were shown to have IC_50_ values of approximately 2.5 μg/mL and 4 μg/mL, similar to each other and the positive control. A one-way ANOVA test to compare these IC_50_ values against that of the negative control (IC_50_ of ~1 µg/mL) showed that both functionally selected clones and the positive control are significantly more resistant to colistin than the negative control (*P* < 0.0005) (Fig. 4B).

Finally, we determined MIC values for colistin (and the related antibiotic polymyxin B) using MIC test strips on agar plates (Fig. 4C). Clones CST10 and CST17 were able to grow in the presence of 3 μg/mL and 4 μg/mL colistin, respectively, similar to the positive control at 4 μg/mL (Fig. 4C). In contrast, growth of the negative control was inhibited by 0.25 μg/mL colistin (Fig. 4C). Similar trends, but with lower MIC values, occurred with polymyxin B, showing resistance across polymyxin classes.

### Expression of functionally selected metagenomic DNA fragments leads to lipid A remodeling consistent with colistin resistance

**Fig 5:**
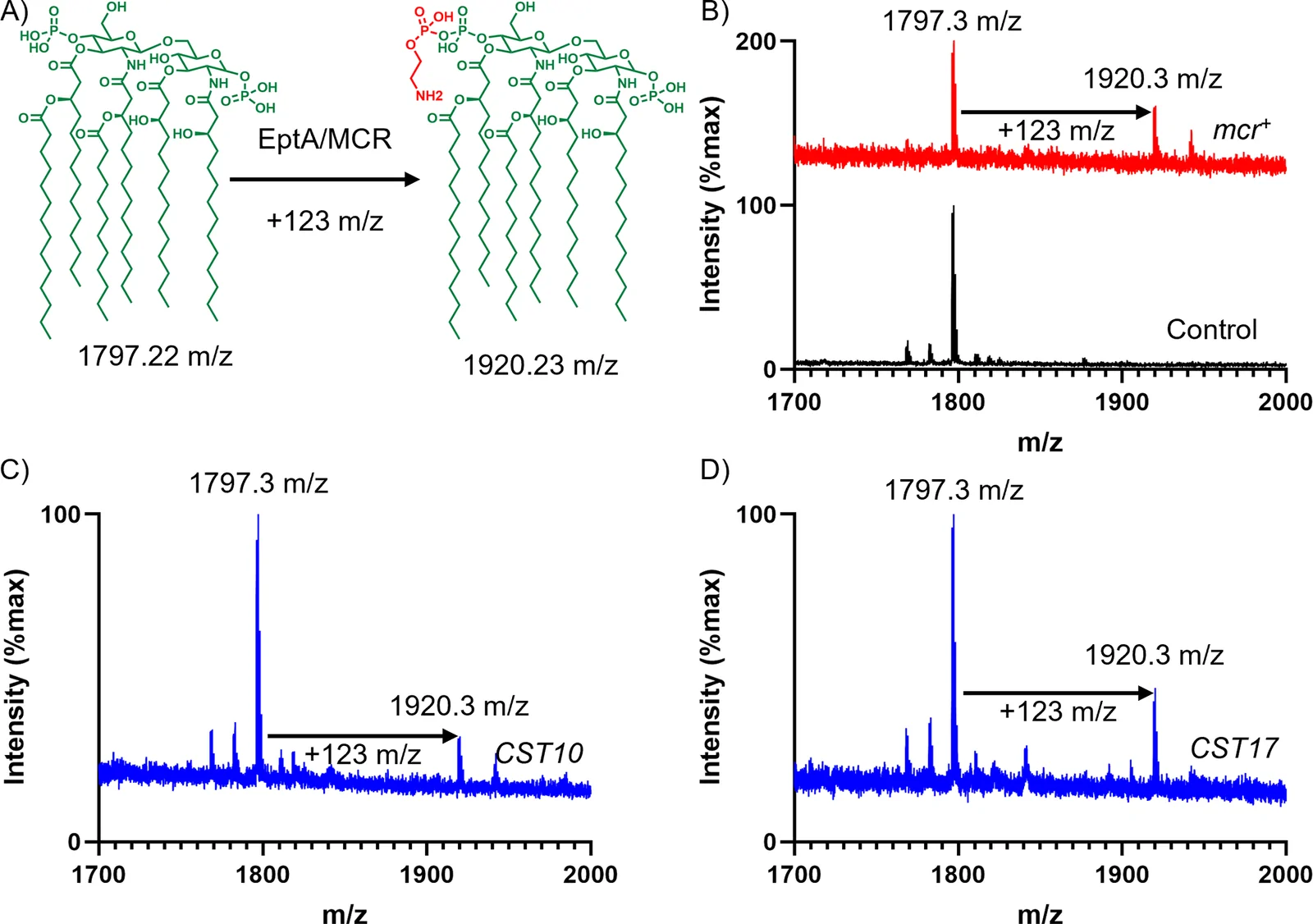
Expression of either mcr or CST genes leads to remodeling of lipid A. (**A**) Molecular reaction catalyzed by EptA and MCR: transfer of a phosphoethanolamine group (red) onto lipid A, resulting in a mass increase of 123 *m/z*. (**B**) Mass spectrometry profile of lipids extracted from *E. coli* containing either an empty plasmid vector (control, black) or a plasmid-expressed mcr gene (mcr+, red). Mass spectrometry profiles of *E. coli* expressing eptA genes from (**C**) CST10 or (**D**) CST17 metagenomic DNA fragments.

EptA and MCR-mediated resistance to colistin in bacteria is due to modification of lipid A in the outer membrane (addition of a phosphoethanolamine moiety) that reduces interaction with the antibiotic (Fig. S1). We set out to verify that the observed colistin resistance in the CST10 and CST17 clones (Fig. 3 and 4) was mediated by this mechanism. Clones CST10 and CST17, as well as the plasmid-only negative control and *mcr-1* positive control, were grown in liquid culture and harvested for their lipid A content. We used matrix-assisted laser desorption/ionization time-of-flight mass spectrometry to quantify lipid A mass changes. The negative control, *E. coli* with an empty vector, produced a lipid species with mass consistent with the expected wild-type 3-deoxy-D-*manno*-octulosonic acid-lipid A (1,797.2 *m/z*) (Fig. 5A and B) (62). In contrast, the *mcr-1*-expressing *E. coli* clone (Fig. 5B), as well as CST10 and CST17 (Fig. 5C and D), contained an additional lipid with a mass increase of ~123 *m/z* (1,920.3 *m/z*), consistent with the addition of a phosphoethanolamine group (Fig. 5A) (62).

### *eptA*17 shows evidence of mobilization within *Acinetobacter*

**Fig 6:**
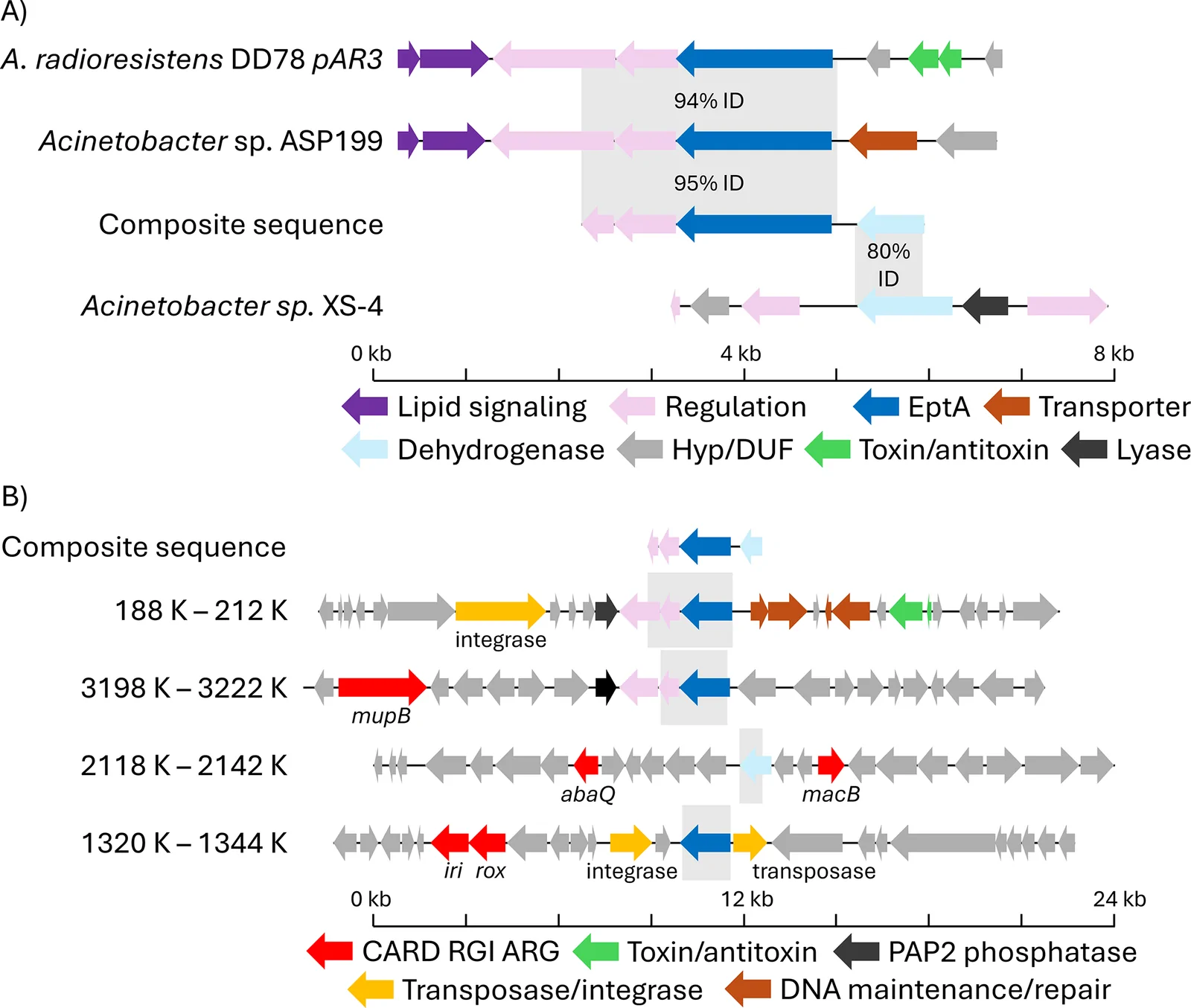
Potential genomic and plasmid native contexts for eptA17. (**A**) Gene schematic representation for a composite sequence of the functionally selected metagenomic DNA fragments (“composite sequence,” center). On the top, gene schematics for genomic (*Acinetobacter* sp. ASP199) and plasmid (*A. radioresistens* DD78 pAR3) sequences with high nucleotide identity and coverage of the eptA-containing 5′ end of the composite sequence. On the bottom, a gene schematic for genomic DNA from Acinetobacter sp. XS-4 with moderately high nucleotide identity and coverage of the 3′ end of the composite sequence containing an alcohol dehydrogenase gene fragment. (**B**) Gene schematic comparing the composite sequence against four regions of the *A. baumannii* strain VB473 genome. Gray boxes indicate regions with 68% (1320 K–1344 K), 69% (3198 K–3222 K), or 89% (2118 K–2142 K, 188 K, and 212 K) nucleotide identity.

Because all the functionally selected colistin-resistant metagenomic DNA fragments appear to have the same chromosomal origin (Fig. 1), we used sequence information from CST16 and CST17 to construct a composite “full length” sequence that encompassed all available information from the 15 inserts. As before (Table S1), this composite sequence has high nucleotide identity (>90%) to *Acinetobacter* species. We noticed, however, that the 5′ end and 3′ end of the composite sequence had the highest identity to different *Acinetobacter* strains according to nucleotide megablast alignment (this held true when we examined just CST17 by itself as well). Specifically, the 5′ end (including the *eptA* gene) aligns best to the chromosome of *Acinetobacter* species ASP199 (~95% identity). This region also showed high sequence identity (~94%) to the pAR3 plasmid from *A. radioresistens* strain DD78 (which contains a toxin/antitoxin gene pair 3′ to the *eptA* region) (Fig. 6A). In both cases, the 3′ region past the *eptA* gene of the composite sequence has essentially no identity locally to either *Acinetobacter* sp. ASP199 or the strain DD78 plasmid. Instead, the 3′ region (containing a fragment of a predicted alcohol dehydrogenase gene) has the highest identity to another *Acinetobacter* strain, sp. XS-4. Here, sp. XS-4 only has local identity to the alcohol dehydrogenase gene (~80% nucleotide identity) and does not encode an *eptA* gene in the vicinity (Fig. 6A).

While our nucleotide megablast alignments found high identity to the genomes and plasmids of *Acinetobacter* sp. ASP199 and *A. radioresistens*, respectively (Fig. 6A), our protein BLAST analysis suggested that the captured EptA protein may come from another lineage (Fig. S5). To attempt to reconcile this observation, we switched to analyzing the composite nucleotide sequence by discontinuous megablast. When we sorted alignments by total coverage, the top hit identified was *A. baumannii* strain VB473 (GenBank CP050388.1). The composite sequence aligned to four regions of the strain VB473 genome (Fig. 6B). Region 1 (188 K to 212 K) contains the *eptA* gene and its two downstream predicted transcriptional regulators, as well as an integrase gene, the PAP2 phosphatase gene, a predicted toxin-antitoxin module, and several genes predicted to function in DNA repair and maintenance. Notably, *mcr* genes are often associated with PAP2 phosphatase genes, which are required for high-level colistin resistance (63). Region 2 (3198 K to 3222 K) also contains the *eptA*, regulatory genes, and PAP2 phosphatase, as well as a Resistance Gene Identifier (RGI) predicted *mupB* mupirocin resistance gene (52, 53). Region 3 (2118 K to 2142 K) contains the alcohol dehydrogenase gene and a pair of predicted antibiotic efflux pumps, *macB* and *abaQ*. Finally, region 4 (1320 K to 1344 K) contains just the *eptA* gene, flanked by integrase and transposase genes, with two nearby predicted rifampin resistance genes (*iri* and *rox*) (Fig. 6B).

## DISCUSSION

We explored, in detail, colistin resistance determinants from a prior 4 μg/mL colistin functional metagenomic selection of a goose microbiome and a new 8 μg/mL colistin selection. All 15 metagenomic gene fragments cloned in this study appear to originate from a single source, based on high nucleotide identity (Fig. S4) and shared gene structure (Fig. 1). Surprisingly, not all the cloned CST constructs supported the same level of growth in the presence of colistin, and some clones displayed MICs below the colistin concentration used in their original functional metagenomic selection (Fig. 3). We hypothesize that these CST constructs likely acquired mutations during or after the cloning process. We were unable to confidently identify mutations during the cloning process due to the suboptimal quality of our sequencing reads (for example, annotation of the original sequencing results implausibly suggested most metagenomic inserts contained highly fragmented open reading frames). Prior studies have demonstrated that carriage of *mcr* genes is associated with a metabolic burden, and optimal antibiotic resistance only occurs after host cells have had an opportunity to adapt through compensatory mutations (64–66) and can revert to susceptibility in the absence of colistin exposure (64). We, therefore, think it is likely that these mutated clones reflect compensation for the metabolic burden of phosphoethanolamine transferase activity. A future experiment cloning these highly similar, but apparently functionally different, *eptA* open reading frames could shed light on the potential balance between phosphoethanolamine transferase activity, metabolic burden, and colistin resistance. As a result, we focused our attention on CST17, which was directly picked from its 8 μg/mL selection, and CST10, which was validated by its ability to confer high levels of colistin resistance (Fig. 4).

Because the colistin resistance-conferring gene fragments came from a small insert functional metagenomic library (the largest captured insert measures approximately 3 kb in length), we were unable to perfectly identify their original host genome. It is highly likely that the origin of this DNA fragment was an *Acinetobacter* bacterium (Table S1), but our analyses suggest that multiple *Acinetobacter* taxa may fulfill this role (Fig. 6 and Fig. S5). The specific local context of the colistin-resistant insert is also unclear, with high nucleic acid identity regions mapping to multiple *Acinetobacter* chromosomal sequences and plasmid sequences (Fig. 6). The homolog-containing plasmid (*A. radioresistens* DD78 plasmid pAR3, NCBI reference sequence NZ_CP038025.1) contains multiple predicted transposases and genes predicted to encode toxin-antitoxin proteins, suggesting mobilization of the plasmid as well as its cargo (Fig. 6A). The plasmid is also annotated as containing genes encoding metal resistance proteins (e.g*., terC*), and the CARD resistance gene identifier tool (53) identified a potential carbapenemase on the plasmid, highlighting that our captured phosphoethanolamine transferase gene may be part of a plasmid conferring resistance to multiple antibiotics and other environmental pressures. Similarly, regions with high nucleotide identity on the genome of *A. baumannii* strain VB473 also contain multiple mobilization-associated genes (integrase and transposase) as well as predicted antibiotic resistance genes (mupirocin, rifampin, macrolides, and quinolones) (Fig. 6B). Notably, this strain of *A. baumannii*, a clinical isolate, has previously been recorded as having resistance to colistin as well as carbapenems (67).

At the sequence, structural, and activity levels, EptA and MCR enzymes are almost indistinguishable (27, 30, 62). Both MCR and EptA enzymes are phosphoethanolamine transferases, and we confirmed the presence of this enzymatic activity in our own strains (Fig. 5C and D). The essential distinction between *eptA* and *mcr* genes is their mobilization, or lack thereof, and their ability to confer colistin resistance (30). The European Society of Clinical Microbiology and Infectious Diseases Committee on Antimicrobial Susceptibility Testing (EUCAST) defines colistin resistance in both *Acinetobacter* and *E. coli* (*Enterobacterales*) as growth in the presence of >2 µg/mL colistin. We found that, in the cases of CST10 and CST17, carriage of the *eptA* gene studied in this manuscript confers colistin resistance past this level in *E. coli* (Fig. 4). This fact, alongside very close relatives of this *eptA* gene having (i) different genomic contexts across *Acinetobacter* taxa, (ii) appearing on a plasmid, and (iii) appearing flanked by mobilization elements (Fig. 6), suggests it may be appropriate to consider it an *mcr* gene or at least a proto-*mcr* gene. One limitation of our study is that, due to the nature of functional metagenomics, we were unable to directly test colistin resistance in the unknown-but-likely *Acinetobacter* sp. host. This means that the assignment of the gene to the *mcr* or *eptA* family must necessarily be provisional.

It has been shown that *Acinetobacter eptA* genes and their regulatory two-component signaling systems can be picked up by and/or induced by transposases, resulting in colistin-resistant bacteria (68–70). This observation parallels the discovery of *mcr*-1 as it was determined that it first emerged on a composite ISApl1 transposon that then transferred to plasmids of pathogenic bacteria (66). While *mcr*-1 was discovered on a swine farm (3), it is hypothesized that it was acquired from a natural environmental source, i.e*.,* the antibiotic resistome (34). Our functional metagenomic selection specifically captured a single *Acinetobacter eptA* gene rather than a collection of *eptA* homologs from other taxa, suggesting that *Acinetobacter* sp., a taxon well known for its high level of horizontal gene transfer and genomic plasticity (71), may be a source of *mcr* genes. This highlights one advantage of functional metagenomics over sequencing-only methods for identifying antibiotic resistance genes, particularly in this case where MCR enzymes are difficult to distinguish from EptA enzymes phylogenetically (Fig. 2).

On the other hand, our approach also comes with limitations. The use of *E. coli* as a heterologous host is one shortcoming as it is possible that other colistin resistance-conferring metagenomic gene fragments could exist in our library but lack proper expression and translation in *E. coli*. Another potential limitation of our approach is that genes that confer colistin resistance when expressed on our library vector may not confer resistance in their native genomic context, putatively an *Acinetobacter* taxon. While colistin resistance in the context of our study may be driven by the plasmid promoter upstream of the metagenomic fragment, it is notable that we found the *eptA*-containing DNA both in positive and negative reading frames with respect to this promoter (Fig. 1). This suggests that in at least one of the cases where we observed growth in the presence of 2 μg/mL colistin (CST14, Fig. 3), transcription was driven more naturally by the *eptA* gene’s native promoter and not the artificial promoter on our vector. Furthermore, the captured *eptA* gene led us to highlight high nucleotide identity homologs that show several hallmarks of mobilization including presence in genomic and plasmid contexts, vicinity to toxin-antitoxin genes, and synteny with transposase and integrase genes. However, this does not necessarily mean that those *eptA* genes are naturally capable of transferring to another host organism. Our functional metagenomic approach suggests that it is possible under the correct circumstances but is not proof.

Migratory birds that can travel long distances are potential vectors of antibiotic resistance genes between antibiotic resistomes and have the potential to disperse resistance genes along their flight path and destination (39, 72–74). Antibiotic resistance genes found in gull feces have displayed evidence of horizontal transfer and diversification throughout the wildlife population, likely facilitated by the abundance of sewage, landfills, and public beaches encountered by gulls (73). Geese, too, have been implicated in being dispersal agents of antibiotic resistance genes through their migration paths (72, 75). In the specific case of *mcr* genes, a study by Cao et al. found that out of a sample of hundreds of birds, approximately 50% carried the *mcr*-1 gene (39). Another study focused on the bar-headed goose in China found 7.3% of fecal samples contained colistin-resistant *E. coli*, often carrying *mcr-1* genes in the context of multidrug-resistant plasmids (74). The sample site location for our functional metagenomic library, part of an urban college campus, suggests that the associated flock of geese would have had a high level of exposure to human activity. The impact of these exposures on the goose gut resistome is not easy to quantify, but the above results and our own highlight the role bird microbiomes play as vectors for antibiotic resistance genes and, in particular, *mcr* or proto-*mcr* genes.

## Data Availability

Sequence data for CST2, CST3, CST4, CST5, CST6, CST7, CST8, CST9, CST10, CST11, CST12, CST13, CST14, CST16, and CST17 are available on GenBank under accession numbers PX673461, PX673462, PX673463, PX673464, PX673465, PX673466, PX673467, PX673468, PX673469, PX673470, PX673471, PX673472, PX673473, PX673474, and PX673475.

## SUPPLEMENTAL MATERIAL

Supplemental material aem.02468-25-s0001.xlsx Fig. S1 to S6; Table S1.

ASM does not own the copyrights to Supplemental Material that may be linked to, or accessed through, an article. The authors have granted ASM a non-exclusive, world-wide license to publish the Supplemental Material files. Please contact the corresponding author directly for reuse.
